# The presence of depression in *de novo* Parkinson’s disease reflects poor motor compensation

**DOI:** 10.1371/journal.pone.0203303

**Published:** 2018-09-19

**Authors:** Yoonju Lee, Jungsu S. Oh, Seok Jong Chung, Jae Jung Lee, Su Jin Chung, Hyojeong Moon, Phil Hyu Lee, Jae Seung Kim, Young H. Sohn

**Affiliations:** 1 Department of Neurology, Yonsei University College of Medicine, Seoul, South Korea; 2 Department of Nuclear Medicine, Asan Medical Center, College of Medicine, University of Ulsan, Seoul, South Korea; 3 Department of Neurology, Ilsan Paik Hospital, Inje University College of Medicine, Goyang, South Korea; 4 Department of Neurology, Myongji Hospital, Goyang, South Korea; 5 Severance Biomedical Science Institute, Yonsei University College of Medicine, Seoul, South Korea; Karolinska Institutet, SWEDEN

## Abstract

Depression frequently accompanies Parkinson’s disease and often precedes the onset of motor symptoms. This study aimed to evaluate the impact of depression on motor compensation in patients with *de novo* Parkinson’s disease. This retrospective cohort study analyzed data from 474 non-demented patients with *de novo* Parkinson’s disease (mean age, 64.6±9.8 years; 242 men) who underwent both dopamine transporter PET scan and depression assessment using the Beck Depression Inventory at baseline. Patients were classified into tertiles by Beck Depression Inventory score. At baseline, high-tertile group (Beck Depression Inventory score ≥15, *n* = 157) showed more severe motor deficits and lower cognitive function than low-tertile group (Beck Depression Inventory score ≤7, *n* = 158, *P* = 0.034 and *P* = 0.008, respectively). Greater motor deficits in high-tertile group than low-tertile group remained significant after controlling for dopamine transporter binding in the posterior putamen, as well as other confounding variables. During follow-up of a median duration of 47 months, high-tertile group received higher levodopa-equivalent doses for symptom control than did low-tertile group after controlling for age, gender, and initial motor deficit severity. These results demonstrate that depression in *de novo* Parkinson’s disease is associated with motor deficit severity at baseline and dose of PD medications during follow-up, suggesting that the presence of depression in *de novo* Parkinson’s disease represents poor motor compensation.

## Introduction

Increasing evidence supports the heterogeneity of Parkinson’s disease (PD) in its clinical presentation and prognosis [1, 2]. Identification of PD subtypes may help understand underlying disease mechanisms, to predict disease course, and to design more efficient personalized therapeutic strategies [3]. Among the potential factors delineating PD subtypes, early burden of non-motor symptoms accompanied in PD has been recognized as an important prognostic marker indicative of poor motor outcomes [4, 5].

Motor symptoms in PD do not develop until approximately half of mesencephalic dopaminergic neurons are lost [6], suggesting the presence of significant motor system compensation. This compensatory ability may reflect an individual’s capacity to tolerate neuropathological lesions, i.e., dopamine depletion in PD, known as neural reserve [7]. Patients with *de novo* PD, who had either olfactory dysfunction or REM sleep behavior disorder, exhibited greater motor deficits than those without these symptoms at similar level of dopamine depletion [8, 9], suggesting that early accompaniment of these non-motor symptoms is associated with reduced compensatory ability in PD.

Depression is another representative non-motor symptom that may precede the onset of motor symptoms [10]: early accompaniment of depression in PD has been proposed to result from the pathological involvement of the monoaminergic nuclei in the brainstem [11]. Accordingly, early accompaniment of depression in *de novo* PD indicates widespread involvement of pathological lesions, which in turn may limit compensatory ability in PD. Despite a previous report showing more physical impairments in depressed patients with early PD compared to non-depressed patients [12], dopamine depletion patterns associated with depression in early PD still remains controversial [13, 14]. To test whether early accompaniment of depression is associated with reduced ability of motor compensation, we analyzed dopamine transporter PET scans and depression levels in patients with *de novo* PD.

## Material and methods

### Study populations

This retrospective cohort study selected subjects from the Yonsei Parkinson Center database (consecutive patients sampled from April 2009 to September 2015) who fulfilled the following selection criteria: (1) had drug-naïve PD; (2) underwent DAT imaging using [^18^F] N-(3-fluoropropyl)-2b-carbon ethoxy-3b-(4-iodophenyl) nortropane (FP-CIT) PET scans; and (3) underwent the Beck Depression Inventory (BDI) assessment. PD in these patients was diagnosed according to the clinical criteria of the UK Brain Bank, the presence of appropriate DAT uptake defects on FP-CIT PET scans, and the presence of PD drug response during a follow-up period >3 months. Part III of the Unified Parkinson’s Disease Rating Scale (UPDRS-motor) was used to assess PD motor symptom severity, while the Mini-Mental State Examination (MMSE) was used to measure cognitive function in each patient at the time of the FP-CIT PET acquisition. Patients who took antidepressants or had an MMSE score < 24 were excluded. We received approval from the Yonsei University Severance Hospital ethical standards committee on human experimentation for this study. Because this study was a retrospective analysis of pre-existing medical data, the need to obtain patient consent was waived.

### Assessment of depression

The BDI was developed to provide a quantitative assessment of depression intensity based on clinical observations, and has been widely used in patients with PD for screening and measuring depression [15]. The Korean version of the BDI has been developed, validated, and used to assess depression in the general population, as well as in patients with PD [16, 17]. Although BDI reliability and validity were demonstrated for assessment of depression in PD, the recommended cut-off point for discriminating between depressed and non-depressed patients with PD varies among studies, ranging from 7–18 points [18]. Leentijens et al. suggested that a single BDI cut-off score to distinguish depressed from non-depressed PD patients is not feasible, because the sum of the sensitivity and specificity was found to change little over a broad range of possible cut-off scores ranging from 6/7 to 16/17 points [19]. Thus, in this study, we classified patients into three tertile groups by BDI score instead of using a single cut-off value. Because BDI score is known to represent depression levels in patients with PD [20], we assumed that high-tertile group encompassed only depressed patients and low-tertile group encompassed only non-depressed patients, while middle-tertile group encompassed a mix of depressed and non-depressed patients.

### Image acquisition and quantitative analyses

To measure striatal DAT binding, we conducted DAT scans using FP-CIT with a GE Discovery STe PET-CT scanner (GE Healthcare Technologies, Milwaukee, WI, USA). In all patients, FP-CIT PET scans were performed in drug-naïve state before PD diagnosis. The details of the PET-CT image acquisition were the same as previously described [8].

Quantitative analyses of FP-CIT PET data were performed following a previously described procedure [8, 21], based on volumes of interest (VOIs): 12 VOIs of bilateral striatal subregions and one occipital VOI were drawn in the same way as described in previous studies. Using DAT concentration in each VOI, DAT binding for each VOI was estimated using the specific/nonspecific binding ratio as a surrogate. This was defined as follows: (mean standardized uptake value of the striatal sub-region VOIs—mean standardized uptake value of the occipital VOI) / mean standardized uptake value of the occipital VOI.

### Longitudinal assessment of the change in levodopa-equivalent dose

After diagnosis of PD, two movement disorder specialists (P.H.L. and Y.H.S.) adjusted the doses of PD medications for effective symptom control at 3- to 6-month intervals. At each visit, the doses of PD medications were checked, and levodopa-equivalent dose (LED) was calculated based on a previously described methodology [22].

### Statistical analyses

Data are expressed as means ± standard deviations. An analysis of variance test with *post hoc* Bonferroni correction was used to compare numeric variables, while a χ^2^ analysis was used to compare non-parametric variables among the three tertile groups. A general linear model was used to compare differences in UPDRS-motor scores among the three groups after controlling for DAT binding in the posterior putamen and other potential confounding variables. A linear mixed model was used to compare rates of longitudinal LED changes among the three tertile groups. Five fixed effects were included in the model: four were between-subject effects (tertile group, age at PD onset, gender, and baseline UPDRS-motor score), and one was a within-subject effect (time). Since most increases in LED occur within the first six months, we regarded time as a categorical variable with a 6-month interval, up to 48 months (when more than 49% of the patients were followed-up). Patients with follow-up durations of 18 months or longer were enrolled in this analysis. The effect of the tertile groups on changes in LED over time was tested with a time × tertile group interaction term, after controlling for age at PD onset, gender, and initial UPDRS-motor score. SPSS Statistics 23 (IBM SPSS, Armonk, NY, USA) was used to perform the statistical analyses. P-values less than 0.05 were considered significant.

## Results

### Baseline demographic and clinical characteristics

A total of 474 patients (mean age, 64.6 ±9.8 years; range, 37–89 years; 242 men) were included in our analysis. The mean symptom duration was 18.3 ± 17.3 months, mean UPDRS-motor score was 21.7 ± 9.4, and mean BDI score was 12.1± 8.3. One hundred fifty-eight patients were in low-tertile group (BDI score ≤7), 159 in middle-tertile group (BDI score, 8–14) and 157 inhigh-tertile group (BDI score≥15). Patients’ baseline clinical and demographic characteristics are shown in Table 1. UPDRS-motor score and MMSE score differed among the three groups (P = 0.025 and P = 0.008, respectively); a *post hoc* analysis indicated that high-tertile group had higher UPDRS-motor scores and lower MMSE scores than did low-tertile group (P = 0.034 and P = 0.008, respectively). Gender distributions tended to differ among the three groups (P = 0.052); more women (55.4%) were in high-tertile group, while more men (58.8%) were in low-tertile group. Other variables, including age and symptom duration, were comparable among the three groups.

**Table 1:** Baseline clinical and demographic characteristics.

Variables		Depression level, tertile			P value
		High (*n* = 157)	Middle (*n* = 159)	Low (*n* = 158)	
Beck Depression Inventory	range	≥ 15	8–14	≤ 7	
	mean	21.4 ± 6.9	10.7 ± 1.9	4.1 ± 2.2	
Age (years)		64.0 ± 9.4	64.6 ± 10.0	65.1 ± 10.0	*0*.*589*
Gender (% women)		55.4	49.7	41.8	*0*.*052*
Symptom duration (months)		18.4 ± 14.9	19.3 ± 19.7	17.1 ± 17.0	*0*.*528*
Mini-Mental Status Examination		27.0 ± 1.9	27.4 ± 1.8	27.6 ± 1.7*	*0*.*008*
UPDRS-motor score		22.8 ± 9.5	22.3 ± 9.1	20.1 ± 9.5*	*0*.*025*

UPDRS-motor, Part III of the Unified Parkinson’s Disease Rating Scale.

Data are means ± SDs unless otherwise indicated.

*, significantly different from high-tertile group by *post hoc* analysis.

### DAT binding and initial motor deficits

**Fig 1:**
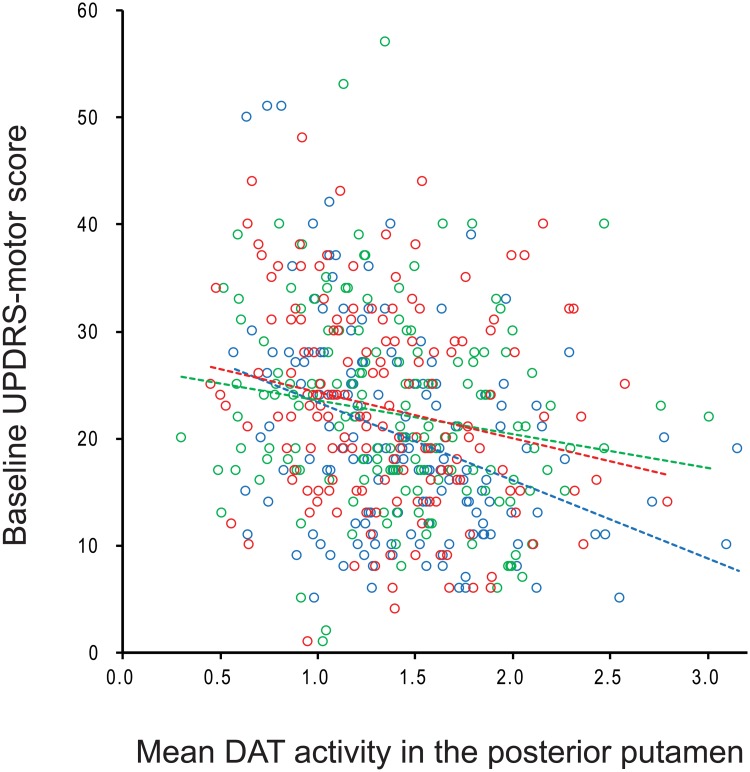
A scatterplot showing baseline UPDRS-motor scores and mean DAT binding in the posterior putamen. High-tertile group (red circles and line) showed higher UPDRS-motor scores at similar DAT binding levels in the posterior putamen than low-tertile group (blue circles and line). Middle-tertile group (green circles and line) showed similar UPDRS-motor scores to high-tertile group. UPDRS-motor, Part III of the Unified Parkinson’s Disease Rating Scale; DAT, dopamine transporter.

DAT bindings in all striatal subregions were similar among the three groups (Table 2). A general linear model showed that high-tertile group had higher UPDRS-motor scores than low-tertile group (*P* = 0.045) after controlling for age, gender, symptom duration, MMSE score, and DAT binding in the posterior putamen (Table 3). However, the interaction effect between patient group and DAT binding in the posterior putamen on UPDRS-motor scores did not differ among the groups (Fig 1).

**Table 2:** Dopamine transporter binding in striatal subregions.

Variables		Depression level, tertile			P value
		High (*n* = 157)	Middle (*n* = 159)	Low (*n* = 158)	
Ventral striatum	Mean	2.23 ± 0.61	2.13 ± 0.60	2.20 ± 0.53	0.242
	Left	2.30 ± 0.63	2.17 ± 0.67	2.24 ± 0.52	0.185
	Right	2.17 ± 0.62	2.08 ± 0.58	2.16 ± 0.56	0.344
Anterior caudate	Mean	2.20 ± 0.70	2.15 ± 0.75	2.19 ± 0.64	0.806
	Left	2.21 ± 0.71	2.15 ± 0.75	2.17 ± 0.63	0.646
	Right	2.18 ± 0.73	2.16 ± 0.77	2.20 ± 0.69	0.894
Posterior caudate	Mean	1.44 ± 0.55	1.44 ± 0.60	1.45 ± 0.58	0.979
	Left	1.40 ± 0.54	1.39 ± 0.60	1.43 ± 0.58	0.768
	Right	1.48 ± 0.60	1.50 ± 0.64	1.47 ± 0.62	0.932
Anterior putamen	Mean	2.26 ± 0.63	2.28 ± 0.70	2.36 ± 0.63	0.329
	Left	2.27 ± 0.66	2.27 ± 0.73	2.35 ± 0.61	0.449
	Right	2.24 ± 0.68	2.29 ± 0.74	2.37 ± 0.72	0.284
Ventral putamen	Mean	1.47 ± 0.43	1.49 ± 0.43	1.54 ± 0.43	0.291
	Left	1.43 ± 0.43	1.43 ± 0.46	1.48 ± 0.41	0.560
	Right	1.50 ± 0.51	1.55 ± 0.50	1.61 ± 0.55	0.217
Posterior putamen	Mean	1.36 ± 0.46	1.40 ± 0.49	1.45 ± 0.48	0.210
	Left	1.36 ± 0.49	1.38 ± 0.56	1.43 ± 0.50	0.445
	Right	1.35 ± 0.56	1.42 ± 0.57	1.47 ± 0.60	0.198

Data are means ± SDs.

**Table 3:** Influence of depression level and dopamine transporter binding in the posterior putamen on UPDRS-motor score.

	Unadjusted		Adjusted*	
	B (S.E.)	P value	B (S.E)	P value
Mean DAT binding	-4.93 (0.88)	<0.001	-4.67 (0.87)	<0.001
The level of depression				
High-tertile	Reference		Reference	
Middle-tertile	-0.24 (1.02)	0.814	-0.26 (1.01)	0.798
Low-tertile	-2.16 (1.03)	0.032	-2.05 (1.02)	0.045

UPDRS-motor, Part III of the Unified Parkinson’s Disease Rating Scale; DAT, dopamine transporter; *B*, estimated slope; S.E., standard error.

*, adjusted for age, gender, symptom duration, and Mini-Mental Status Examination score.

### Longitudinal changes in levodopa-equivalent dose

**Fig 2:**
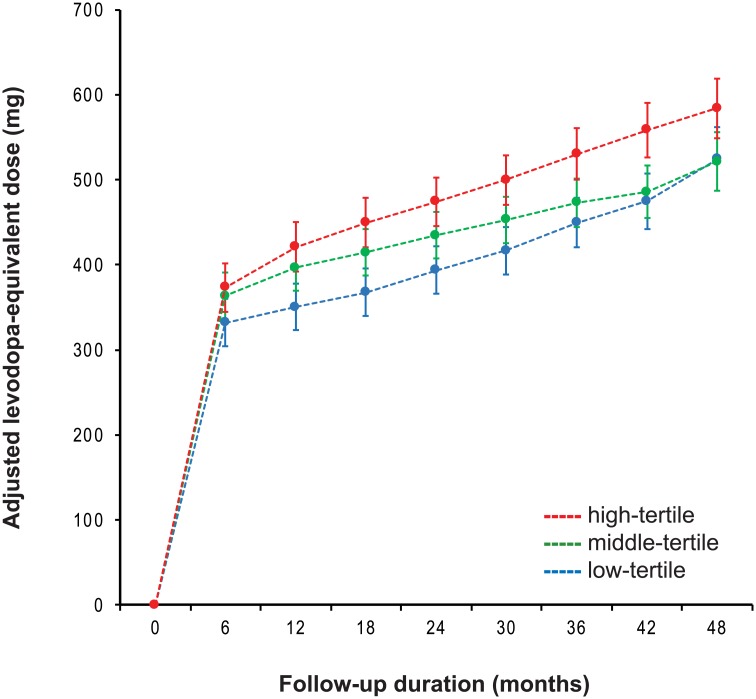
Longitudinal increases in levodopa-equivalent doses. High-tertile group received higher levodopa-equivalent doses for symptom control than low-tertile group. Middle-tertile group received lower LEDs than high-tertile group after follow-up of three years or longer. There was no significant interaction between the tertile groups and time in the mixed model.

The median follow-up duration was 47 months (range, 4 to 107 months). There was no significant interaction between the tertile groups and time in the mixed model (*P* = 0.193), indicating that the pattern of longitudinal changes in LED did not differ among the tertile groups. However, high-tertile group required higher LEDs for symptom control compared to low-tertile group over the follow-up period. Middle-tertile group received lower LEDs than high-tertile group after follow-up of three years or longer (Table 4, Fig 2).

**Table 4:** Levodopa-equivalent doses during follow-up.

Time	Depression level, tertile			Overall P value^a^	Post-hoc P value^b^		
	High	Middle	Low		High vs. Low	High vs. Middle	Middle vs. Low
**0**	0	0	0		NS	NS	NS
**Month 6**	373.32 (14.72)	363.62 (13.79)	332.03 (14.11)		0.129	NS	0.328
**Month 12**	421.16 (14.72)	396.78 (13.79)	350.50 (14.05)		0.002	0.680	0.056
**Month 18**	449.67 (14.72)	414.56 (13.84)	367.71 (14.05)	Group: < 0.001	< 0.001	0.247	0.053
**Month 24**	474.25 (14.72)	434.76 (13.79)	393.82 (!4.10)	Time: < 0.001	< 0.001	0.151	0.114
**Month 30**	499.61 (14.90)	452.92 (13.79)	416.93 (14.21)	Group × Time: 0.193	< 0.001	0.065	0.208
**Month 36**	530.38 (15.22)	473.26 (14.53)	449.51 (14.72)		< 0.001	0.020	0.752
**Month 42**	558.72 (16.41)	485.72 (15.76)	474.90 (16.81)		0.001	0.004	NS
**Month 48**	584.12 (17.79)	521.41 (17.36)	524.51 (18.92)		0.007	0.004	NS

NS, not significant.

Data are means (standard error).

^a^*P*-values calculated by linear mixed model analysis.

^b^Bonferroni correction *P*-values of the post-hoc comparison.

## Discussion

This study demonstrated that depressionin *de novo* PD is associated with initial motor deficit severity: high-tertile group exhibited more severe motor deficits than did low-tertile group, even after controlling for DAT binding in the posterior putamen, as well as other potential confounding variables. In addition, high-tertile group received higher LEDs to control PD symptoms, compared with low-tertile group, during the follow-up period after controlling initial motor deficit severity and other potential confounding variables. These results suggest that the presence of depression in *de novo* PD indicates poor motor compensation at baseline, which in turn is maintained during follow-up period. On the contrary to our initial expectation, the amounts of striatal DAT depletion weresimilar among the three groups.

Our classification of patients based on BDI-score tertiles might be ambiguous. However, the cut-off score (≤7) for the low-tertile group was approximate to the lowest cut-off score proposed in previous studies of PD and depression [19, 20]. In a previous study, this cut-off showed a negative predictive value of 1.00 [19], which suggested that the low-tertile group in this study reasonably represented non-depressed patients with PD. The cut-off score for the high-tertile group was the same as the cut-off score proposed previously in other studies [23]. This previous cut-off previously estimated a specificity of 0.93 [19]. Additionally, because the pooled prevalence of clinically relevant depression in PD outpatient settings was approximately 40% [24], the high-tertile group may well represent depressed patients with PD.

Previous studies repeatedly showed an inverse correlation between depression score and striatal DAT binding [13, 25, 26]. However, Ceravolo et al. demonstrated that this inverse relationship was no longer observed in patients with *de novo* PD [14]. The present results, as well as those in the latter study, suggest that depression in *de novo* PD does not require striatal dopamine depletion. Pathological involvement in the raphe nucleus and locus coeruleus, which occurs prior to PD motor symptoms, according to pathological PD staging by Braak et al. [27], might be responsible for premotor occurrence of depression in PD [10]. A previous study using transcranial sonography demonstrated that reduced echogenicity in the raphe nucleus (suggestive of structural change) was associated with a history of depression prior to PD onset [28], which supports the aforementioned assumption. In the present study, greater motor deficits were observed in depressed patients, compared with non-depressed patients, despite similar levels of striatal dopamine depletion, which again supports the hypothesis that non-dopaminergic lesion contributes to greater motor deficits in depressed patients.

Previous longitudinal studies have shown that the presence of depression represents a more rapid decline in cognitive and motor function in patients with PD [29, 30], although a longer PD duration in depressed than in non-depressed patients in these studies obscure whether different PD stages might influence their results rather than the presence of depression. Ravina et al. analyzed a pooled sample of 413 patients with early untreated PD, and found that baseline depression level was a significant predictor for worsening UPDRS scores of activities of daily living [12]. This study failed to show more rapid motor progression in depressed patients compared to non-depressed patients, due to similar pattern of longitudinal changes in LED. However, the maintenance of higher LEDs in depressed patients compared to non-depressed patients during 48-month follow-up, even after controlling initial motor deficit severity, is somewhat in line with findings of previous studies [12, 29, 30], which indicates that poor motor compensation at baseline in depressed patients is maintained during follow-up.

DAT scans and all baseline assessments were performed in drug-naïve patients that sufficiently excluded the influence of PD medications on these assessments. Nevertheless, this study has some notable limitations. First, some of the depressed patients in this study might convert to non-depressed status at follow-up, which has been previously observed [31]. Thus, initially depressed patients who are persistently depressed at follow-up might differ from those whose depression goes into remission. Second, a recent report by Fereshtehnejad *et al*. showed that non-motor features such as dysautonomia, cognitive impairment, and REM sleep behavior disorder could be important indicators for malignant rapid progressive subtype of PD [2]. Therefore, the association between depression and motor deficit severity shown in the present study might be confounded by these non-motor features. Third, this study was a retrospective cross-sectional study, a design that is inadequate for evaluating disease progression. A future study with prospectively designed longitudinal follow-up is needed to confirm whether depressed patients with *de novo* PD represent poor motor outcome.
